# Decapeptide from Potato Hydrolysate Induces Myogenic Differentiation and Ameliorates High Glucose-Associated Modulations in Protein Synthesis and Mitochondrial Biogenesis in C2C12 Cells

**DOI:** 10.3390/biom12040565

**Published:** 2022-04-11

**Authors:** Yi-Ju Chen, Rathinasamy Baskaran, Ching Fang Chang, Zuhair M. Mohammedsaleh, Wan-Teng Lin

**Affiliations:** 1 Department of Surgery, Taichung Veterans General Hospital, Taichung 40704, Taiwan; chenyiju5668@gmail.com; 2 Department of Animal Science and Biotechnology, Tunghai University, Taichung 40704, Taiwan; 3 Department of Bioinformatics and Medical Engineering, Asia University, Taichung 413305, Taiwan; baskaran@asia.edu.tw; 4 Department of Food Science, Tunghai University, Taichung 40704, Taiwan; g08621006@thu.edu.tw; 5 Department of Medical Laboratory Technology, Faculty of Applied Medical Sciences, University of Tabuk, Tabuk-71491, Saudi Arabia; zsaleh@ut.edu.sa; 6 Department of Hospitality Management, College of Agriculture, Tunghai University, Taichung 40704, Taiwan

**Keywords:** sarcopenia, peptide, muscle atrophy, mitochondrial biogenesis, myogenesis

## Abstract

Sarcopenia is characterized as an age-related loss of muscle mass that results in negative health consequences such as decreased strength, insulin resistance, slowed metabolism, increased body fat mass, and a substantially diminished quality of life. Additionally, conditions such as high blood sugar are known to further exacerbate muscle degeneration. Skeletal muscle development and regeneration following injury or disease are based on myoblast differentiation. Bioactive peptides are biologically active peptides found in foods that could have pharmacological functions. The aim of this paper was to investigate the effect of decapeptide DI-10 from the potato alcalase hydrolysate on myoblast differentiation, muscle protein synthesis, and mitochondrial biogenesis in vitro. The treatment of C2C12 myoblasts with DI-10 (10 µg/mL) did not induce cell death. DI-10 treatment in C2C12 myoblast cells accelerates the phosphorylation of promyogenic kinases such as ERK, Akt and mTOR proteins in a dose-dependent manner. DI-10 improves myotubes differentiation and upregulates the expression of myosin heavy chain (MyHC) protein in myoblast cells under differentiation medium with high glucose. DI-10 effectively increased the phosphorylation of promyogenic kinases Akt, mTOR, and mitochondrial-related transcription factors AMPK and PGC1α expression under hyperglycemic conditions. Further, decapeptide DI-10 decreased the expression of Murf1 and MAFbx proteins, which are involved in protein degradation and muscle atrophy. Our reports support that decapeptide DI-10 could be potentially used as a therapeutic candidate for preventing muscle degeneration in sarcopenia.

## 1. Introduction

Sarcopenia is the primary cause of physical disability, poor quality of life, and death in older persons. It is characterized by a loss in skeletal muscle mass and function without any underlying disease [1]. It is the most common cause of physical frailty in older people, with prevalence varying from 5% to 50% in those aged 60 and up, depending on age [2]. Sarcopenia in aged people causes muscle protein degradation, often surpassing protein production [3]. Sarcopenia is also linked to other conditions such as diabetes, nonalcoholic fatty liver disease, and cardiovascular disease, which include high blood pressure and arterial stiffness [4]. As a result, sarcopenia care is critical for healthy aging. Despite these attempts, no FDA-approved medication for sarcopenia has been developed to date. With the worldwide adult population over 60 predicted to reach 2 billion by 2050, there has been a surge in interest in creating sarcopenia diagnostic tools and medications to improve quality of life and decrease healthcare costs [5].

The diminished regeneration potential of skeletal muscle stem cells appears to be the cause of sarcopenia or muscle atrophy. Understanding the proliferation and differentiation mechanisms of muscle stem cells during muscle regeneration is therefore crucial for the development of treatment options for sarcopenia and other muscular degenerative diseases [6]. By activating the mammalian target of rapamycin (mTOR) and inactivating glycogen synthase kinase 3 (GSK3), IGF-PI3K-Akt signaling promotes skeletal myotube hypertrophy [7]. The 70-kDa ribosomal protein S6 kinase is then phosphorylated by mTOR, which promotes protein synthesis. Furthermore, Akt suppresses protein degradation via Forkhead box O (FOXO)-mediated proteasome activity by inactivating GSK3b; hence, boosting protein translation via eIF-2B. mTOR complex 1 (mTORC1) is a critical step in the development of muscle hypertrophy by boosting protein synthesis [8]. It is activated in response to growth hormones, nutrition, and increased mechanical strain. Mitochondrial dysfunction and alterations in mitochondrial mass and structure are linked to the development of sarcopenia. There is an age-related decrease in mitochondrial mass, which could be due to the dysfunction of mitochondrial biogenesis master regulators such as AMPK and PGC-1 [9,10]. There are age-dependent abnormalities in mitochondrial structure in addition to decreasing mass, suggesting an imbalance between mitochondrial fusion and fission [11].

Many studies have shown that muscle atrophy during aging is mainly due to increased protein breakdown through the ubiquitin–proteasome pathway [12,13,14]. In particular, ubiquitin–protein ligases (E3S), atrogin-1/MAFbx, and MuRF-1 play a central role in muscle atrophy [15,16]. Atrogin-1/MAFbx and MuRF-1 expression in the early stages of the atrophy process lead to muscle mass loss [17]. Mitochondrial biogenesis is enhanced in response to a metabolic shift, such as energy requirements increasing the oxidative stimulus [18]. Skeletal muscle, a crucial component in the regulation of energy metabolism, processes up to 75% of insulin-stimulated glucose disposal by translocating GLUT4 to the plasma membrane in response through the activation of the AMP-activated protein kinase (AMPK) pathway [19]. The myotube is a metabolically active cell type that produces myogenes after differentiation from a myoblast or a pre-existing myotube. This differentiation process imposes metabolic demand, which triggers mitochondrial biogenesis via the coordinated expression of nuclear and mitochondrial genes, which is controlled by a network of transcription factors including PGC-1 α, NRF-1, and TFam [20]. Peroxisome proliferator-activated receptor γ coactivator 1α (PGC1α), is one of the gate keeper genes that activate mitochondrial biogenesis by many nuclear transcriptional regulators including Nrf-1 and mitochondrial transcription factor A (TFam) [21].

It is generally recognized that aging is linked to a lack of appetite, which can lead to a reduction in food consumption, protein-energy deficiency, and weight loss. As a result, it is indeed necessary to test various dietary approaches to determine that individuals with sarcopenia get enough nourishment to maintain their muscle mass [22]. Bioactive peptides, which are mostly produced from hydrolyzed proteins, are physiologically active compounds. Soybean and other plant-derived peptides have been found to have a variety of pharmacological actions, including anti-hypertension, anti-cancer, cholesterol-lowering, and anti-obesity effects [23]. Bioactive peptides extracted from soybeans also lowered cardiovascular disease risk factors [24]. Potatoes are a good source of dietary protein, minerals, and antioxidants. Potato protein hydrolyzate (PPH) has been found to protect the intestinal mucosa from ethanol-induced damage due to its antioxidant action [25]. PPH was discovered to be beneficial in lowering diabetes-related heart and liver damage by inhibiting apoptosis and stimulating mitochondrial biogenesis by restoring SIRT1 expression that had been reduced by a high-fat diet [26]. The effect of decapeptide DI-10 (DIKTNKPVIF: D—Aspartic acid; I—Isoleucine; K—Lysine; T—Threonine; N—Asparagine; K—Lysine; P—Proline; V—Valine; I—Isoleucine; F—Phenylalanine) isolated from PPH on skeletal muscle protection, on the other hand, is yet to be determined. In this study, the effect of DI-10 treatment on skeletal muscle atrophy and high glucose-induced myogenic aversions such as myogenic differentiation, protein synthesis initiation and mitochondrial biogenesis is investigated.

## 2. Materials and Methods

### 2.1. Chemicals

All of the chemicals used in this study were of analytical grade and were purchased from Sigma-Aldrich (St. Louis, MO, USA). Peptide DI-10 (DIKTNKPVIF: D-Aspartic acid; I-Isoleucine; K-Lysine; T-Threonine; N-Asparagine; K-Lysine; P- Proline; V-Valine; I-Isoleucine; F-Phenylalanine) used in the study was commercially synthesized by DG peptides Co., Ltd., China (Hangzhou, China) was about 98% purity.

### 2.2. Cell Culture

C2C12 murine myoblast cells was purchased from Bioresource Collection and Research Center (BCRC, Taiwan) were cultured in Dulbecco’s Modified Eagle Medium (DMEM) with 10% Fetal Bovine Serum (FBS) containing antibiotics streptomycin (100 μg/mL) and penicillin (100 U/mL) in a 10 cm culture plate. Cell culture plate was maintained at 37 °C in a humidified CO_2_ incubator with a 5% CO_2_ supply. Cells were subcultured at 80% confluence and media was changed once in 2 days. For the high-glucose challenge, appropriate amounts of D-glucose were added to the culture media.

### 2.3. MTT Assay

MTT assay was used to evaluate the effect DI-10 on C2C12 cell viability, and 1 × 10^4^ cells were seeded in a 96-well plate in 200 μL of DMEM and allowed to attach overnight in CO_2_ incubator. Then, the cells were treated with different concentrations of DI-10 (2, 2.5, 5, 7.5 and 10 µg/mL) in DMEM and incubated for 24 h. For high glucose experiments, C2C12 cells were cultured in DMEM to a 70–80% confluence with growth medium containing 5.5 mM D-glucose (5.5 mM, control) and 24.5 mM D-mannitol. The high glucose challenge groups were supplemented with an additional 5, 15 or 30 mM of D-glucose for 24 h. Then, cells were washed with PBS thrice and replaced with the fresh DMEM containing different concentration of DI-10 (2, 2.5, 5, 7.5 and 10 µg/mL). After 24h the medium was aspirated, 20 μL of MTT (5 mg/mL) were added and incubated at 37 °C for 4 h. The purple formazan crystals formed were dissolved in 200 μL of dimethyl sulfoxide and absorbance was read at 540 nm.

### 2.4. Differentiation of C2C12 Cells

To induce differentiation in C2C12 myoblasts to myotubes, 4 × 10^5^ cells the cells were grown in normal growth medium (DMEM supplemented with 10% FBS). Once the cells reach 80–90% confluence, DMEM was removed from the cells, washed thrice with PBS and replaced with differentiation medium (DMEM with 2% horse serum). The medium was replaced every 2 days and cells were allowed to differentiate for 6 days. To examine the effects of DI-10 on myogenic differentiation, DI-10 (0, 5, 7.5 and 10 µg/mL) was added to the medium.

### 2.5. Western Blotting Analysis

Western blotting was performed following methods reported previously [27]. Cells were collected at the end of treatment period and washed in PBS twice, then lysed using lysis buffer (CelLytic^TM^ MT Cell Lysis Reagent-Sigma) with protease inhibitor cocktail (PIC) on ice for 1 h. Cell lysates were then centrifuged for 15 Min at 13,000 rpm at 4 °C. Protein concentration in the sample was quantified by Lowry’s method. Using SDS–PAGE (8–12%), equal amounts (40 μg) of protein were separated by electrophoresis and subsequently blotted to nitrocellulose membrane. Membrane was blocked with 5% skimmed milk in TBST at room temperature for 1 h, washed thrice with TBST and incubated overnight with specific primary antibody at 4 °C. Then, the membrane was washed with TBST thrice and incubated with HRP conjugated secondary antibody for 1 h at room temperature. Protein bands were visualized using enhanced chemiluminescence (ECL) horseradish peroxidase (HRP) substrate (Millipore, CA, USA) and images were acquired using iBright FL1500 imaging system (Invitrogen, Carlsbad, CA, USA) and analyzed with ImageJ software (version 1.4.3.67) (NIH, Bethesda, MD, USA), respectively.

### 2.6. Statistical Analysis

The results shown are the means ± SD of three independent experiments. Statistical analysis was performed using a one-way analysis of variants using SPSS 16 software (SPSS, Chicago, IL, USA).

## 3. Results

### 3.1. Effect of DI-10 on Myoblast Cell Viability

**Figure 1:**
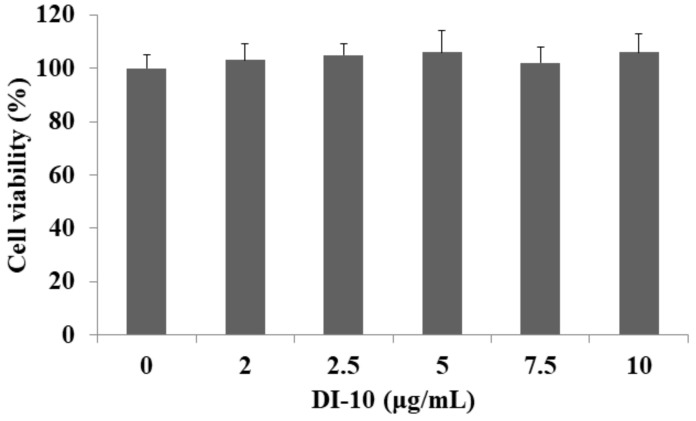
Effect of decapeptide DI-10 on myoblast cell viability. C1C12 cells were treated with different concentration of DI-10.

MTT cell viability assay was used to determine the cytotoxic effect of DI-10 on C2C12 myoblast cells. For this, C2C12 cells were treated with different concentrations of the decapeptide DI-10 (0, 2.5, 5, 7.5, and 10 μg/mL). Up to 10 μg/mL, no significant cell death was observed, suggesting no toxic effect of DI-10 on myoblast cells (Figure 1).

### 3.2. DI-10 Activates ERK, Akt, mTOR, and FOXO3a Proteins and Facilitates Protein Synthesis in Myoblast Cells

**Figure 2:**
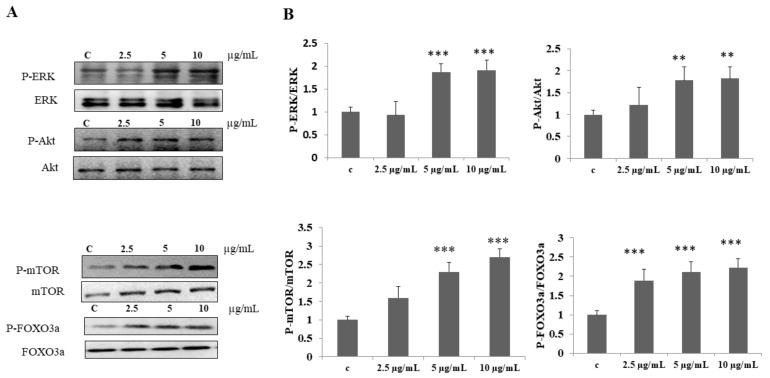
DI-10 activates ERK, Akt, mTOR, and FOXO3a proteins and facilitates protein synthesis in myoblast cells. Dose-dependent activation of ERK, Akt, mTOR, and FOXO3a was observed in myoblast cells after DI-10. (**A**) Western blotting show phosphorylation of ERK, Akt, mTOR, and FOXO3A in C2C12 cells after DI-10 treatment. (**B**) Graph showing densitometric analysis. *** *p* < 0.001, and ** *p* < 0.01 compared to control group.

The effect of decapeptide DI-10 on cell survival and protein synthesis pathways was examined. Phosphorylation of ERK, Akt, mTOR, and FOXO3a proteins were quantified using Western blot analysis. The dose-dependent activation of ERK, Akt, mTOR, and FOXO3a was observed in myoblast cells after DI-10 treatment (Figure 2). Low-dose (2.5 μg/mL) DI-10 treatment does not significantly increase the phosphorylation of ERK, Akt, and mTOR. However, 5 and 10 μg/mL of DI-10 significantly increase the phosphorylation of ERK, Akt, mTOR, and FOXO3a proteins, suggesting that DI-10 peptides have an effect in regulating protein synthesis in muscle cells.

### 3.3. DI-10 Improves the Viability of C2C12 Cells under High Glucose Stress

**Figure 3:**
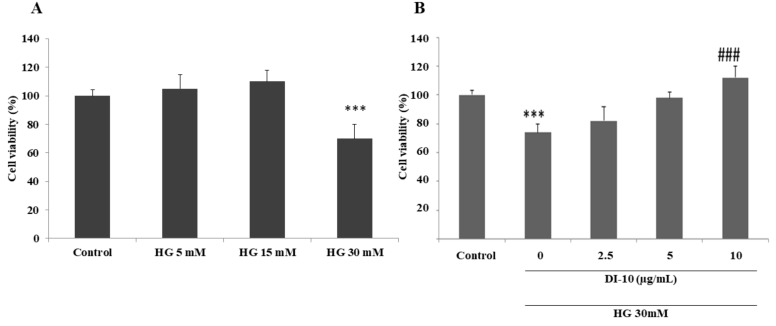
DI-10 improves the viability of C2C12 myoblast cells under high glucose stress. (**A**) C2C12 cells were treated with different concentrations of D-glucose (5, 15, and 30 mM) and the cell viability was determined with MTT assay. (**B**) C2C12 cells were treated with 30 mM HG followed by different concentrations of DI-10. *** *p* < 0.001 compared to control groups and ### *p* < 0.001 compared to the high glucose.

Aging-associated conditions such as diabetes are very common in aging, we have used high glucose to model diabetic conditions in C2C12 cells.C2C12 cells were treated with different concentrations of D-glucose (5, 15, and 30 mM) and the cell viability was determined with MTT assay. D-glucose at 5 and 15 mM concentrations does not induce significant cell death, however, at 30 mM D-glucose 70.4% cell viability was observed and used for our further studies. To determine the cytoprotective effect of decapeptide DI-10, C2C12 cells were treated with high glucose (30 mM) for 24 h followed by different concentrations of DI-10 (5, 7.5, and 10 μg/mL). Concentrations of 5, and 7.5 μg/mL DI-10 did not significantly increased the cell viability in HG treated cells. However, 10 μg/mL of DI-10 significantly increased the cell viability in HG treated C2C12 cells (Figure 3).

### 3.4. Decapeptide DI-10 Promotes Myoblast Differentiation under High-Glucose Conditions

**Figure 4:**
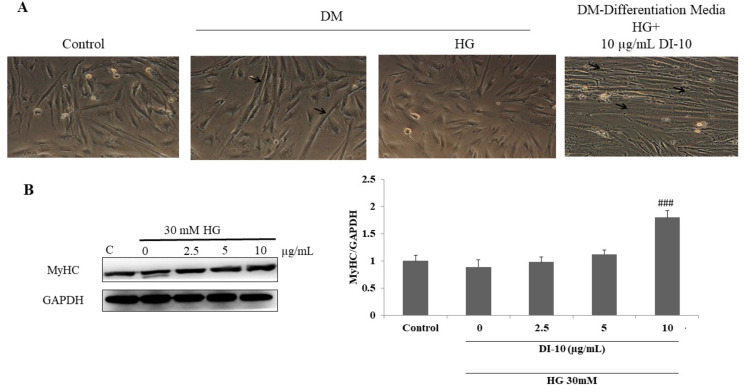
Decapeptide DI-10 promotes myoblast differentiation under high-glucose conditions. Effect of decapeptide DI-10 on myogenic differentiation under hyperglycemic conditions was determined by (**A**) microscopic observation of cell morphology. Photomicrographs were obtained using an Olympus^®^ CKX53 microscope (100×) (**B**) MyHC expression in C2C12 cells under HG conditions. ### *p* < 0.001 compared to the high glucose.

The effect of the decapeptide DI-10 on myogenic differentiation under hyperglycemic conditions was determined by a microscopic observation of cell morphology and MyHC expression (Figure 4). DI-10 treatment at 10 μg/mL induces structural changes in C2C12 cells from flat-spindle shape to long myotubes with thick spindle shape. Further, DI-10 significantly increased the expression of MyHC protein, suggesting myoblast differentiation efficacy of DI-10.

### 3.5. Effect of DI-10 on Akt, mTOR, and AMPK Phosphorylation under Hyperglycemic Conditions in C2C12 Cells

**Figure 5:**
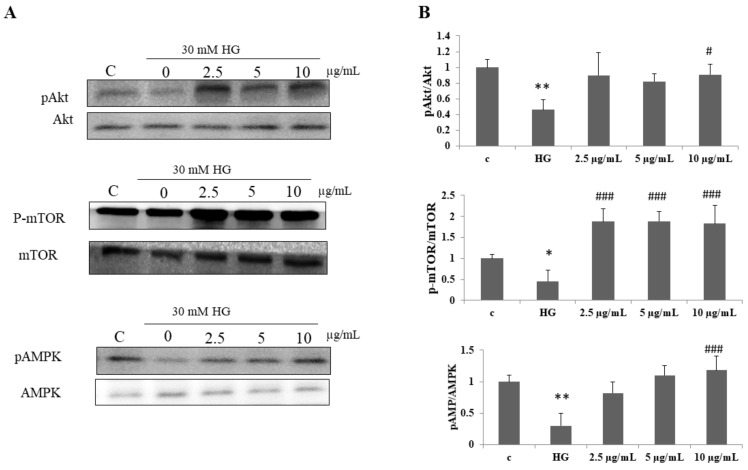
Effect of DI-10 on Akt, mTOR, and AMPK phosphorylation under hyperglycemic conditions in C2C12 cells. (**A**) Immuno blot showing phosphorylation of Akt, mTOR, and AMPK proteins involved in protein synthesis. (**B**) Graph showing densitometric analysis. ** *p* < 0.01 and * *p* < 0.05 compared to control group and # *p* < 0.05 and ### *p* < 0.001 compared to the high glucose.

Then, we examined the activation of Akt, mTOR, and AMPK protein involved in protein metabolism and myogenic differentiation (Figure 5). The phosphorylation of Akt, mTOR, and AMPK was significantly reduced in high glucose-treated cells. DI-10 at 10 μg/mL concentration significantly increased the phosphorylation of Akt and AMPK. However, all doses of DI-10 (2.5 and 5 μg/mL) significantly increased the phosphorylation in C2C12 cells under high-glucose conditions.

### 3.6. DI-10 Inhibits Muscle Atrophy under Hyperglycemic Conditions through Modulating MAFbx and MuRF1

**Figure 6:**
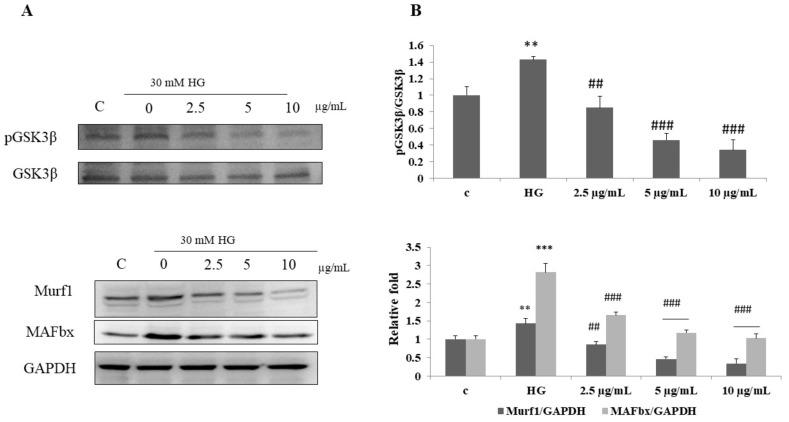
Effect of DI-10 on the protein involved in muscle atrophy. (**A**) DI-10 inhibits muscle atrophy under hyperglycemic conditions through modulating pGSK3β, MAFbx and MuRF1. (**B**) Graph showing densitometric analysis. *** *p* < 0.001 and ** *p* < 0.01 compared to control group and ### *p* < 0.001 and ## *p* < 0.01 compared to the high glucose.

Ubiqueligase E3, such as atrogin-1(MAFbx) and muscle ring-free finger-1 (Murf-1), is known to contribute toward the breakdown of proteins which was regulated by GSK-3β. So, we evaluated the effect of DI-10 on the protein involved in muscle atrophy (Figure 6). In C2C12 myoblast cells under hyperglycemic conditions, the expression of pGSK3β, MAFbx and MuRF1 was found to be increased significantly. DI-10 significantly decreased the expression of pGSK3β, MAFbx and MuRF1 under high-glucose conditions in a dose-dependent manner.

### 3.7. DI-10 Improves the Expression of Mitochondrial Biogenesis-Regulating Factors in Myotubes in High-Glucose Conditions through NRF-1 and TFam

**Figure 7:**
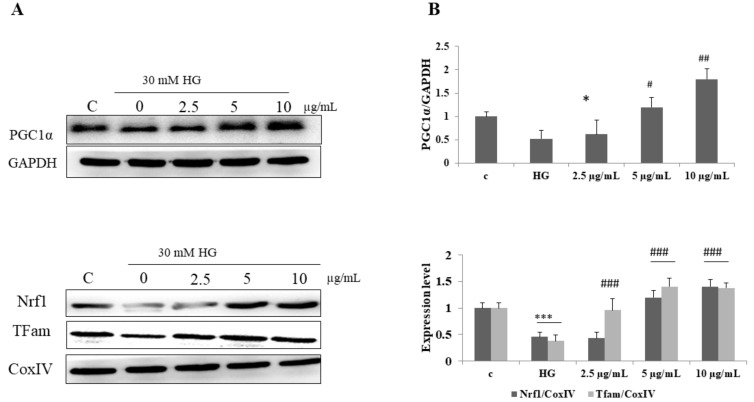
Effect of DI-10 on mitochondrial biogenesis pathway protein expression under high-glucose conditions. (**A**) DI-10 improves the expression of mitochondrial biogenesis-regulating factors in myotubes in high-glucose conditions through NRF-1 and TFam. (**B**) Graph showing densitometric analysis. *** *p* < 0.001 and * *p* < 0.05 compared to control and ### *p* < 0.001, ## *p* < 0.01 and # *p* < 0.05 compared to the high glucose.

In our present study, the expression of mitochondrial biogenesis-regulating factors PGC1α, NRF-1 and TFAM were decreased significantly in high-glucose conditions. DI-10 significantly increased the expression of PGC1α, NRF-1 and TFAM under high-glucose conditions in dose-dependent manner (Figure 7).

## 4. Discussion

Epidemiological research reports reveal that protein consumption is linked to muscle mass retention. Many studies show that increased protein or essential amino acids consumption, and the branched-chain amino acid leucine, along with resistance exercise, can help reduce fiber atrophy in sarcopenic muscle by influencing both anabolic and catabolic pathways [28]. Previous studies have shown that bioactive peptides from potato hydrolysates have therapeutic potential against various disease conditions such as diabetes, hypertension, and obesity-related cardiac and skeletal muscle injuries [29,30,31]. However, it is well known that certain diets and nutrition, such as essential amino acids, milk-based proteins, creatine monohydrate, essential fatty acids, and vitamin D, when combined with resistance exercise can further enhance the beneficial effects on muscle mass and strength in the elderly [32]. Supplementing with amino acids following high-resistance exercise training has a synergistic impact on the contraction-induced increase in muscle protein synthesis [33,34]. Amino acids have a function in the phosphorylation of translation proteins through an mTOR-mediated mechanism, according to human studies [33,35].

It has been observed that the reciprocal relationship and activation of the ERK, Akt, and mTOR pathways are essential for effective myoblast differentiation. FOXO (forkhead box O) is a key transcription factor linked to longevity regulation, and the overexpression of FOXO effectively prevents the p-62 buildup in the muscle cells due to aging [36,37]. In different aging models, IGF1 and Akt signaling pathways are shown to have a key role in both aging and aging-associated muscle loss via FOXO transcriptional activity. In support of sarcopenia models, drugs have been discovered that induce FOXO phosphorylation as a possible treatment agent for sarcopenia patients [38]. In our present study, DI-10 increases myoblast differentiation by activating ERK, Akt, and mTOR signaling, and its promyogenic action is reliant on the phosphorylation of ERK and Akt. Further, it also increases the p-FOXO3a expression in a dose-dependent manner. A previous report by Sandri et al. showed that FOXO activation could induce muscle atrophy in mice, which is contradictory to our present findings [39]. The fact that variable levels of FOXO activation can increase stress resistance or rather cell death might explain why FOXO activity can be beneficial or harmful during muscle aging [40]. While normal FOXO activation could help maintaining protein homeostasis and muscle performance, excessive activation can result in impaired muscle function attributable to the protein hyperactivation of the protein degradation pathway.

Most of the protein degradation in muscle cells is carried out by proteolytic molecules such as calpain, proteasomes, and lysosomes. Desmin and dystrophin are key proteins specific to muscles that are sensitive to this protease activity, although alpha-actinin, tropomyosine, and phylamine are generally insensitive [41]. During sarcopenia, ubiqueligase E3, such as atrogin-1 and muscle ring-free finger-1 (Murf-1), is known to contribute toward the breakdown of proteins. The activation of the ubiquitin–proteasome system, which is linked to autophagy, is associated with increased protein degradation and decreased protein synthesis during sarcopenia [17].

Mitochondrial biogenesis demands coordinated changes in the expression of a wide range of metabolic genes encoded by both genomic and mitochondrial DNA (mtDNA). Furthermore, a complicated transcriptional network controls protein expression. One of the most important transcription activators in this network is mitochondrial transcription factor A (TFam) [42,43]. TFam is primarily responsible for mtDNA replication and transcription [44]. TFam has the potential to be important in mitochondrial biogenesis. Nuclear respiratory factors (NRF) NRF-1 and -2 are essential molecules that directly regulate the expression of TFam in both humans and rodents [20,45]. The results from our study show the DI-10 elevated TFam expression in muscle cells treated with high glucose, which is in line with the previous reports of Cederroth et al. where mice fed with the diet containing high levels of soybean isoflavones, the expression of genes associated with mitochondrial metabolism, including TFAM, was dramatically elevated in the muscles [46].

**Figure 8:**
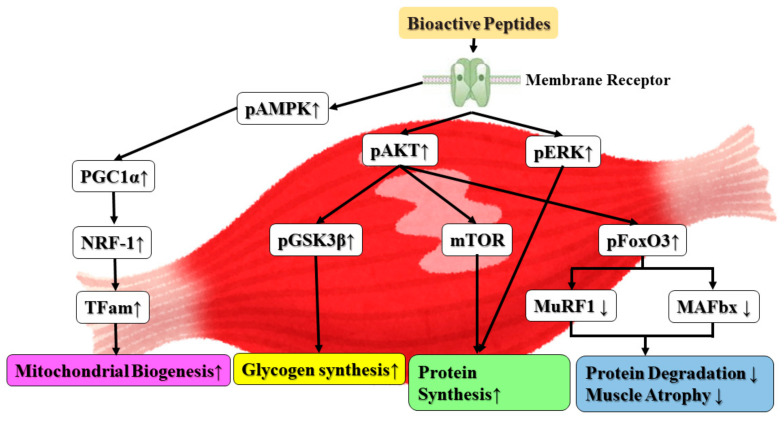
Bioactive peptides enhance myogenic differentiation and skeletal muscle protein synthesis under high-glucose conditions in C2C12 myoblast cells. ↑ increase, ↓ decrease.

In conclusion, we demonstrated that decapeptide DI-10 increases mitochondrial biogenesis in C2C12 myoblasts. Regarding the molecular mechanism, PGC1α targeted was mediated through both NRF-1/TFam dependent pathways. In our present study, we focus on the effect of DI-10 in stressful conditions such as diabetes, which is very common in aging. Our results show that DI-10 reversed the effects of hyperglycemia in skeletal muscle by activating protein synthesis through the Akt/mTOR pathway and preventing protein degradation by downregulating Murf-1 and MAFbx through FoxO3 (Figure 8). Thus, decapeptide DI-10 from potatoes could be a powerful agent in nutritional intervention for age-associated muscular dysfunctions.

## Data Availability

The data that support the findings of this study are available upon request from the corresponding author.
